# The Work of the Holy Ghost

**Published:** 1887-05-14

**Authors:** 


### Words of Consolation.

[Communications for this column should be addressed, "Chaplain," care of the Editor of The Hospital, who will gratefully welcome any suggestions or inquiries from matrons, nurses, and the staff generally.]

XXXIII.—The Work of the Holy Ghost.

"He shall convince the world of sin, and of righteousness, and of judgment" (John xvi, 8). Think how these convictions may follow one another. If by the help of the Holy Spirit we are shown how sinful we are, and so come to believe that we are by nature miserable and naked and unfit for Heaven; then comes the great question pressing with awful force upon the soul, "What shall we do to be saved?" How shall we be accounted righteous, or become righteous? Thank God, the Holy Spirit does not leave us only convinced of sin. He goes on to show us how by faith in Christ we may not only be accounted righteous, but also become righteous. He convinces us that we may through Christ be justified, and how, remaining in a state of grace, we may utterly abolish the whole body of sin, and become at last perfectly holy. If He were to convince us of sin, and then leave us alone to bewail our wretchedness, we should be hopeless and heartbroken indeed. But he does not give us a broken and contrite heart without pointing to the way of salvation almost at the same time. We say almost, not always quite, at the same time: for He does make some people very miserable and sad about themselves (and well that He should) before He allows them to grasp the comfort which belongs to more matured faith. He *does* convince of sin before He allows a full conviction of righteousness. He does show certain souls how bad they are, or have been, before He allows them to feel certain, or very hopeful, about salvation. And this is really very merciful dealing with those who are becoming poor in spirit. A vast majority of Christians like to believe themselves on the road to heaven before they have ever really admitted and yielded to convictions of their own sinfulness. They have never known any deep sorrow for sin, because they have stifled first convictions. When, for instance, you find this kind of spirit, "I have had my faults, but I have never done anything very wrong: so I am not much concerned about my salvation, nor fear but what all will come right for me in the end by the mercy of God." Ah! that is taking righteousness for granted before there has been any real conviction of sin permitted. This is a very dangerous condition, indicating that the work of the Holy Spirit has never been allowed a fair beginning. To those in whom a godly sorrow has been wrought not to be repented of, the feeling has come so well expressed in the confession, "The remembrance of them is grievous unto us, the burden of them is intolerable." Blessed are they who have not merely confessed, but felt as much. The temptation to choke convictions of sin is very common; nowhere more common, we suspect, than among the convalescent after they have been listening in sickness for many days to the still small voice as it pleaded with them for repentance.

(*To be continued.*)

